# Metatarsophalangeal joint extension changes ultrasound measurements for plantar fascia thickness

**DOI:** 10.1186/s13047-018-0267-0

**Published:** 2018-05-29

**Authors:** Michael J. Granado, Everett B. Lohman, Keith E. Gordon, Noha S. Daher

**Affiliations:** 1 School of Allied Health Professions, Loma Linda University, Loma Linda, CA 92350 USA; 2 Department of Physical Therapy, Loma Linda University, Loma Linda, CA 92350 USA; 3 Physical Therapy and Human Movement Sciences, Northwestern University, Chicago, IL USA

**Keywords:** Fasciitis, Fasciosis, Fasciopathy, Windlass, Toe dorsiflexion, Ultrasonography, Treatment

## Abstract

**Background:**

Ultrasound is an inexpensive method for quantifying plantar fascia thickness, especially in those with plantar fasciitis. Ultrasound has also been used to assess the effectiveness of various treatments for plantar fasciitis by comparing plantar fascia thickness before and after an intervention period. While a plantar fascia thickness over 4 mm via ultrasound has been proposed to be consistent with plantar fasciitis, some researchers believe the 4 mm plantar fascia thickness level to be a dubious guideline for diagnosing plantar fasciitis due to the lack of standardization of the measurement process for plantar fascia thickness. In particular, no universal guidelines exist on the positioning of the metatarsophalangeal (MTP) joints during the procedure and the literature also has inconsistent protocols. The purpose of this study is to investigate and compare the influence of MTP joint extension on plantar fascia thickness in healthy participants and those with unilateral plantar fasciitis.

**Methods:**

The plantar fascia thickness of forty participants (20 with unilateral plantar fasciitis and 20 control) was measured via ultrasound three times at three different MTP joint positions: 1) at rest, 2) 30° of extension from the plantar surface, and 3) maximal extension possible.

**Results:**

The plantar fascia became significantly thinner as MTP joint extension increased in both the plantar fasciitis group (*p* < 0.001) and the control group (*p* < 0.001). In the plantar fasciitis group, the involved plantar fascia was 1.2 to 1.3 mm thicker (p < 0.001) than the uninvolved side depending on the MTP joint position. In the control group, the difference in plantar fascia thickness between the two sides was less than 0.1 mm (*p* < 0.92) at any MTP joint position.

**Conclusions:**

MTP joint position can influence the ultrasound measurement of plantar fascia thickness. It is recommended that plantar fascia thickness measurements be performed with the toes at rest. If MTP joints must be extended, then the toes should be extended maximally and then noted to ensure subsequent ultrasound procedures are repeated. Standardizing the position of the MTP joints is not only important for attaining the most accurate thickness measurement of the plantar fascia, but is also important to researchers who use plantar fascia thickness to determine the effectiveness of various plantar fasciitis interventions.

## Background

The plantar fascia is a flat band of connective tissue residing in the sole of the foot with attachments from the medial tubercle of the calcaneus to the proximal phalanges [1]. If excessive and repetitive tensile forces are imposed onto the plantar fascia, presumed development of microtrauma results in a condition known as plantar fasciitis [2, 3]. In the United States, approximately one million outpatient visits for plantar fasciitis were made annually during 1995–2000 [4]. It was also estimated that in 2007, the cost to treat plantar fasciitis was between 192 and 376 million US dollars [5]. Despite the pervasiveness of this condition, no gold standard exists for diagnosing plantar fasciitis, although the diagnosis is often made through a clinical history and physical examination backed by imaging [6, 7].

Ultrasound is a widely used tool especially in conjunction with plantar fasciitis because it provides an inexpensive, and noninvasive method for quantifying the plantar fascia with accuracy levels comparable to magnetic resonance imaging (MRI) [8, 9]. Several studies agree that a plantar fascia thickness over 4 mm via ultrasound is consistent with plantar fasciitis [10–14]. However, some researchers have argued that a lack of standardization in the measurement process for plantar fascia thickness makes it challenging to properly validate the 4 mm reference guideline [7, 15]. One critical component for standardizing measurement procedures is a thorough characterization of the relationship between plantar fascia thickness and toe position.

Several studies that have demonstrated an increase in plantar fascia tension [16–18] and a rise in the medial longitudinal arch [17, 19] as a result of metatarsophalangeal (MTP) joint extension. In addition, Garcia et al. [20] who found that MTP joint extension results in an increase in plantar soft tissue stiffness along with a concomitant decrease in overall plantar soft tissue thickness. Cumulatively, these studies strongly suggest that MTP joint extension could influence measures of plantar fascia thickness. Yet, guidelines for positioning the MTP joint during ultrasound measurements of the plantar fascia have not been developed. For example, the European Society of Musculoskeletal Radiology has produced its procedural recommendations for how the plantar fascia thickness should be measured [21], but a recommendation for how the toes should be positioned during the examination is noticeably absent. While many ultrasound studies measuring plantar fascia thickness either leave the toes in a resting position or do not even indicate the position of the toes during the examination, other authors have advocated extending the toes to improve the border definition of the plantar fascia during the procedure [11, 15, 22–24]. However, it is unclear if doing so alters the acquired ultrasound measurements.

The purpose of this study was to investigate the influence of active MTP joint extension on plantar fascia thickness. Since the ultrasound procedure is often performed in those with plantar fasciitis, a comparison between plantar fasciae in those with unilateral plantar fasciitis with healthy control participants was also conducted in the study. More specifically, the objectives of this study were: 1) examine the changes in plantar fascia thickness by MTP joint extension position and side (i.e., involved versus uninvolved) in the plantar fasciitis group; 2) examine changes in plantar fascia thickness by MTP joint extension position and side (right versus left) in the control group; and 3) compare changes in plantar fascia thickness by MTP joint extension position in the uninvolved side of the plantar fasciitis group with the control group. We hypothesized that the thickness of the plantar fascia would decrease in both groups studied as MTP joint extension is increased.

## Methods

### Participants

All participants signed an informed consent, and the study was approved by the Loma Linda University Human Research Participant Protection (HRPP) Program/Institutional Review Board (Approval No. 5150186**)**. Participants with plantar fasciitis were required to exhibit the classic symptoms of plantar fasciitis (i.e., plantar heel tenderness, morning pain with the first few steps out of bed) and have had symptoms persisting longer than 6 weeks to ensure participants were not in an acute phase of the condition. Individuals were excluded from the study if they had any neurologic, systemic inflammatory, metabolic, connective tissue, or inner-ear disorders. Those with severe toe deformities, trauma/surgery to the lumbar spine or lower extremities, an antalgic gait pattern, a cortisone injection over the preceding three months, or recent consumption of balance-altering medication were also excluded from the study.

### Measurement of plantar fascia thickness

**Fig. 1:**
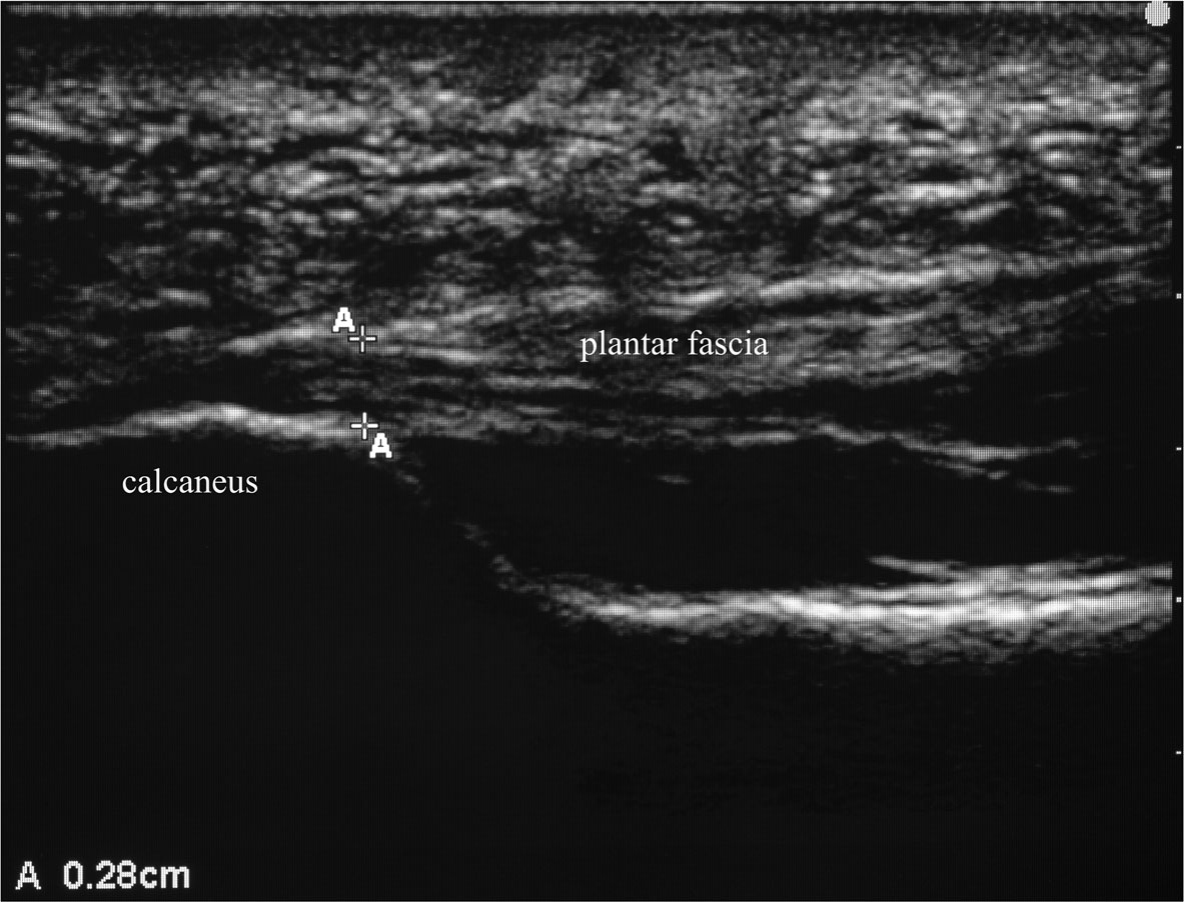
Longitudinal sonogram of the plantar fascia with the thickness being measured at the anterior margin of the calcaneus

Sagittal thickness of the plantar fascia was measured with a 13–6 MHz linear array transducer (Sonosite M-Turbo Ultrasound System, Bothell, WA, USA) and acoustic coupling gel applied onto the plantar surface of the heel. Participants were positioned in prone with the examined foot over the edge of the examination table and the ankle in neutral. The transducer was positioned over the plantar surface of the heel approximately 0.5 cm medial to the midline longitudinal axis of the foot in order to visualize a longitudinal view of the plantar fascia. The thickness of the plantar fascia was then measured at the anterior margin of the calcaneus (Fig. 1).

**Fig. 2:**
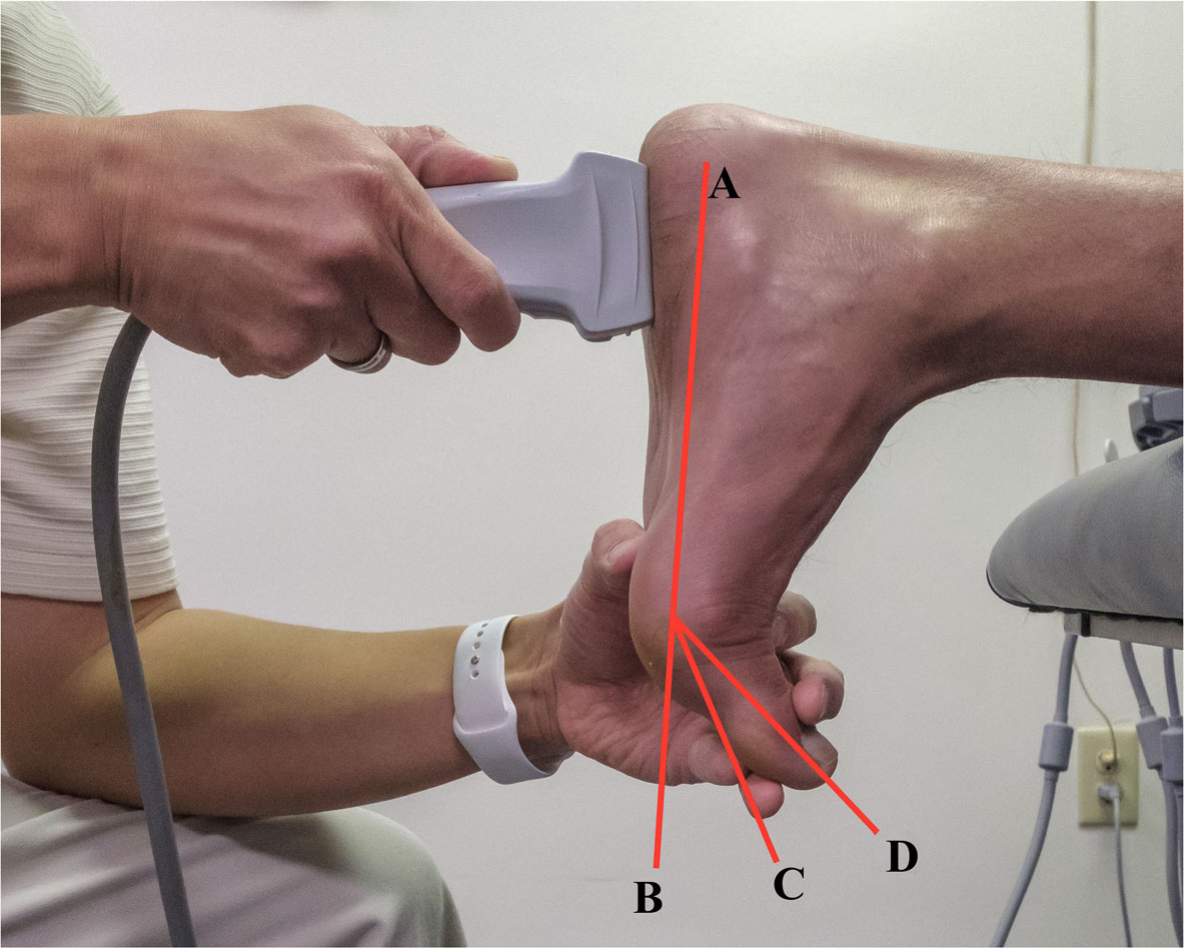
An illustration of the three metatarsophalangeal (MTP) joint extension positions employed during the ultrasound measurement for plantar fascia thickness: (**a**) plantar surface of the foot; (**b**) at rest; (**c**) 30° relative to the plantar surface of the foot; (**d**) max extension possible

The ultrasound measurement was performed with the toes in three different MTP joint positions: 1) at rest, 2) 30° of active extension from the plantar surface, and 3) maximal extension actively possible by the participant (Fig. 2). All of the toes were extended passively together to the desired position by the examiner whereby the participant was then asked to actively hold the position while the examiner continued to monitor for any movement. Goniometry was only performed when the MTP joints were extended to 30° as measurement was not necessary in the at rest or max extension positions. Measurement of first MTP joint extension was performed on the medial aspect of the foot with the proximal arm of the goniometer parallel with the plantar surface of the foot and the distal arm aligned with the midline of the proximal phalanx of the first toe. The traditional method for measuring extension at the first MTP joint involves aligning the proximal arm of the goniometer with first metatarsal, either dorsally or over the medial surface of the foot rather than the plantar surface of the foot [25]. A modification was employed in an effort to make comparisons between feet from different individuals more reliable as outlined by Allen and Gross [26]. In the foot-flat position, the MTP joints are typically extended 20° from the midline of the metatarsals [27]. However, it was felt that different foot types could result in the first metatarsal having a variable position. The plantar surface of the foot being fixed would not suffer from this inconsistency and would allow for a “standard” position in between the two other MTP joint positions (i.e., at rest and at max extension). When at rest and at max extension, a specific joint angle was not necessary, which was why goniometry was not performed. Ultrasound measurements conducted with the MTP joints at rest were intended to place the least amount of tension onto the plantar fascia, whereas maximal MTP joint extension was necessary to apply the most amount of tension. A standard MTP joint position during the initial and final conditions of ultrasound measurements would had most likely resulted in relative inaccurate tissue tension in some participants (i.e., presence of unwanted plantar fascia tension at the initial position or inadequate plantar fascia tension when the MTP joints were extended maximally).

The ultrasound examinations were performed by one licensed physical therapist with sixteen years of clinical experience and who had completed several continuing education radiology courses. However, the examiner had no previous experience in using ultrasound for imaging purposes. Crofts et al. [28] demonstrated that relatively new ultrasound examiners with minimal, but structured training could still acquire reliable ultrasound data. Thus prior to the study, the examiner met with a certified ultrasound technician for four instructional sessions over the course of one month to become familiar with the equipment and technique. During the study, the examiner was blind to which foot was afflicted with plantar fasciitis when measuring plantar fascia thickness in participants. As well, the side first to be examined was randomly selected in each participant with a flip of a coin. Ultrasound measurements were performed three times at each MTP joint position. Measuring plantar fascia thickness has been found to be more reliable when the mean of three ultrasound measurements was used rather than a single measurement [22, 24, 28].

### Data analysis

Data was analyzed using SPSS Statistics Software version 24.0 (IBM Corp, Armonk, NY). Mean ± standard deviation (SD) was computed for quantitative variables and frequencies (%) for categorical variables. Normality of continuous variables was assessed using Shapiro-Wilk test and box plots. A 2 × 3 repeated factorial analysis of variance (ANOVA) was conducted to examine the effect of side (involved vs. uninvolved) and MTP joint extension position (at rest vs. 30° vs. max) on plantar fascia thickness (mm) in the plantar fasciitis and control groups. To compare changes in plantar fascia thickness by MTP joint extension position in the uninvolved side between the plantar fasciitis group and control group, 2 × 3 mixed factorial ANOVA was used. The level of significance was set at *p* ≤ 0.05.

## Results

The study involved 40 healthy participants (20 with unilateral plantar fasciitis and 20 control) between the ages of 18 and 65 years, all of whom had a body mass index (BMI) below 35 kg/m^2^. The mean ± SD age of the participants was 44.8 ± 12.2 years and BMI 26.8 ± 4.5 kg/m^2^. The majority were also females (*n* = 26, 65%, see Table 1). In the plantar fasciitis group when analyzing the uninvolved and involved sides by MTP joint extension position, the results from the 2 × 3 repeated ANOVA showed that there was a significant difference in mean ± SD plantar fascia thickness (mm) among the different positions, at rest vs 30° vs max, (4.6 ± 0.13 vs. 4.3 ± 0.13 vs. 4.2 ± 0.13, F_2,38_ = 62.2, η^2^ = 0.77, *p* < 0.001), as well as between involved and uninvolved side (see Table 2.) However, there was no significant interaction between side and position (F_2,38_ = 0.90, *p* = 0.41). Bonferroni post hoc comparisons showed that mean plantar fascia thickness differed significantly between at rest and 30°, at rest and max, and 30° and max (*p* < 0.001).

**Table 1:** Mean (SD) of general characteristics by group at baseline

	Plantar fasciitis group (*n* = 20)	Control group (*n* = 20)
Female, *n* (%)	13 (65%)	13 (65%)
Age	47 (11.9)	43 (12.6)
BMI	28.3 (4.3)	25.3 (4.3)

Abbreviations: *SD* standard deviation, *BMI* body mass index

Units: Age, years; BMI, kg/m^2^

**Table 2:** Mean (SD) plantar fascia thickness (mm) at each MTP joint position by group type

	Plantar fasciitis group (*n* = 20)				Control group (*n* = 20)			
MTP Joint Extension Position	Involved	Uninvolved	Difference (95% CI)	*p*-value^a^ (η^2^)	Right	Left	Difference (95% CI)	*p*-value^b^ (η^2^)
At rest	5.2 (1.1)	3.9 (0.7)	1.3 (0.8–1.8)	< 0.001 (0.60)	3.4 (0.5)	3.4 (0.4)	< 0.1 (0.1–0.2)	0.92 (0.00)
30°	4.9 (1.0)	3.7 (0.6)	1.2 (0.8–1.7)		3.2 (0.4)	3.2 (0.4)	< 0.1 (0.08–0.1)	
Max	4.8 (1.0)	3.6 (0.7)	1.2 (0.7–1.8)		3.0 (0.4)	3.0 (0.4)	< 0.1 (0.1–0.2)	
*p*-value^c^ (η^2^)	< 0.001 (0.77)				< 0.001 (0.75)			

Abbreviations: *SD* standard deviation, *MTP* metatarsophalangeal, *CI* confidence interval, η^2^ effect size

^a^Involved vs uninvolved

^b^Right vs left

^c^MTP joint extension position

In the control group when comparing both sides by MTP joint position, there was a significant difference in mean ± SD plantar fascia thickness among the three different positions (3.4 ± 0.01 vs. 3.2 ± 0.01 vs. 3.0 ± 0.01, F_2,38_ = 56.1, η^2^ = 0.75, *p* < 0.001; see Table 2) and Bonferroni post hoc comparisons showed that mean plantar fascia thickness differed significantly between at rest and 30°, at rest and max, and 30° and max (*p* < 0.001). However, there was no significant difference in mean ± SD plantar fascia thickness between right and left side (3.2 ± 0.04 vs. 3.2 ± 0.03, F_1,19_ = 0.01, *p* = 0.92), and no significant interaction between position and side (F_2,38_ = 0.24, *p* = 0.79).

When comparing the uninvolved side from the plantar fasciitis group with the average of two sides from the control group, a significant difference was found in mean ± SD plantar fascia thickness between the two study groups (3.7 ± 0.04 vs. 3.2 ± 0.04, F_1,38_ = 9.85, η^2^ = 0.21, *p* = 0.003). A significant difference in mean ± SD plantar fascia thickness among the three different positions (3.7 ± 0.06 vs. 3.5 ± 0.06 vs. 3.3 ± 0.06, F_2,76_ = 60.4, η^2^ = 0.61, *p* < 0.001) was also found with post hoc comparisons showing mean plantar fascia thickness differed significantly between at rest and 30°, at rest and max, and 30° and max (*p* < 0.001). However, there was no significant interaction between position and group (F_2,76_ = 0.69, *p* = 0.50).

## Discussion

In 1993, the first report of ultrasound being used to measure plantar fascia thickness was published [10]. Since then, ultrasound has become an important tool for not only visualizing the plantar fascia, but is also used to assess the effectiveness of various treatments for plantar fasciitis [14, 29, 30]. However, the lack of standardization for measuring plantar fascia thickness with ultrasound may make the process more challenging by affecting the accuracy of results. The main concept to ascertain from this study is that as MTP joints are actively extended, the plantar fascia decreases in thickness when observed during ultrasound. The current practice for measuring plantar fascia thickness via ultrasound does not involve a standardized position for the toes. Thus, the MTP joint position can vary depending on the preference of the examiner causing the thickness measurements to potentially vary as well. Ultimately, there is some evidence to suggest a need to re-examine how plantar fascia thickness is measured. Based upon the results from this study, the MTP joint position should be standardized during the ultrasound procedure, either at rest or at max extension to ensure ease of reproducibility. While keeping the toes at rest is most likely the easiest position to replicate and should be the established position when measuring plantar fascia thickness, some clinicians prefer to extend the toes in order to improve the border definition via ultrasound [11, 15, 22–24]. Since the methodology lacks standardization, it is the recommended that the toes be at rest during the ultrasound measurement for best reproducibility and only at max MTP joint extension when improved visibility of the plantar fascia border is necessary. The position of the MTP joints during the procedure should always be recorded to guarantee consistent protocols are followed in subsequent ultrasound measurements. For example, researchers studying the efficacy of a particular treatment intervention for plantar fasciitis would want to ensure that any change in plantar fascia thickness is due to the intervention and not because of inconsistent toe positioning during the ultrasound procedure.

The average plantar fascia thickness in healthy participants that has been reported in the literature is between 2.6 and 3.9 mm [8, 10, 13, 22, 31–34]. This relatively large range in normative values is most likely due to the discrepancies within the current methodology and participant variation [22]. For instance, Bisi-Balogun et al. [24] measured the thickness along different locations of the plantar fascia and found that the mean and ± SD could vary between 2.26 ± 0.4 mm to 3.06 ± 0.6 mm. This is important because the location along the plantar fascia where its thickness is measured has always lacked consistency in the literature [7, 15, 24, 32]. Further complicating the matter is that different segments of the plantar fascia are susceptible to further thickness variations due to gender and body weight characteristics [15]. This has even prompted some authors to suggest comparing the thickness of symptomatic plantar fasciae with contralateral asymptomatic feet in those with unilateral plantar fasciitis rather than compare to a standardized threshold of 4 mm [7]. McMillan et al. [14] conducted a systematic review and meta-analysis and found that participants with chronic plantar fasciitis had a plantar fascia thickness that was about 2.2 mm more than the corresponding control participants. In our study, the plantar fascia thickness of the involved side in the plantar fascia group was significantly higher when compared to the uninvolved side with a difference of 1.2 to 1.3 mm depending on the MTP joint position. One possible reason for the slightly lower difference in contrast to the McMillan et al. study could be because the MTP joint extension position was carefully controlled in our study and not in the other studies analyzed in the McMillan et al. meta-analysis.

**Fig. 3:**
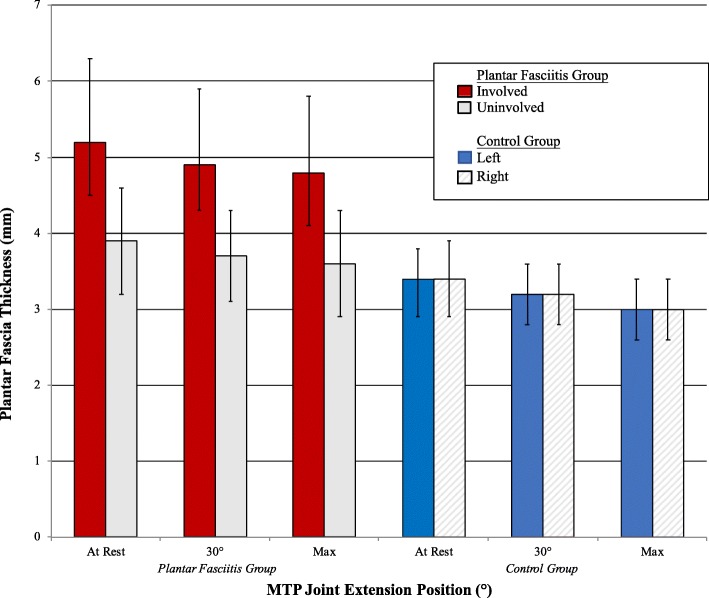
Mean ± SD of plantar fascia thickness (mm) by MTP joint extension position, side, and group. Abbreviations: SD, standard deviation; MTP, metatarsophalangeal

When analyzing the plantar fascia thickness of those in the control group, the two sides were not significantly different (Table 2). But when plantar fascia thickness of the control group was compared to the uninvolved side in the plantar fasciitis group, the uninvolved plantar fascia was still significantly thicker (see Table 2). The literature has been inconclusive on the difference between asymptomatic plantar fasciae in those with unilateral plantar fasciitis and healthy individuals. Some studies have reported that the asymptomatic plantar fasciae in those with unilateral plantar fasciitis were thicker [11, 35], whereas other studies have reported no significant difference between the two groups [10, 13]. In this study, the asymptomatic plantar fasciae were thicker than the controls at every MTP joint extension position (Fig. 3). The former may be an indication of either an unhealthy compensatory response during gait or an inherent biomechanical flaw that predisposed these individuals with unilateral plantar fasciitis to have increased plantar fascia thickness on the asymptomatic side. Bilateral plantar fasciitis has been reported to be present in 13 to 30% of the cases so it is reasonable to see a slightly thicker asymptomatic plantar fascia in those with unilateral plantar fasciitis as a potential harbinger [36, 37].

A limitation of this study was that the examiner was not blind to the position of the MTP joints during the ultrasound procedure. Controlling for this potential bias would be a welcome addition in future studies. As well, it would be of strong interest to assess how MTP joint extension would affect plantar fascia thickness on MRI. Observing a similar relationship on MRI to the ultrasound results in this study would further highlight the need for MTP joint position to be standardized during the ultrasound procedure.

## Conclusions

Based upon the findings from this study, the amount of MTP joint extension can strongly influence the ultrasound measurement of plantar fascia thickness and should be taken into account during the procedure. It is recommended that plantar fascia thickness measurements be performed with the toes at rest. If MTP joints must be extended, then the toes should be extended maximally and then noted to ensure subsequent ultrasound procedures are repeated. Standardizing the position of the MTP joints is not only important for attaining the most accurate thickness measurement of the plantar fascia, but is also imperative to researchers who use plantar fascia thickness to determine the effectiveness of various plantar fasciitis interventions.
